# Follitropin Beta as a Rescue Protocol for Poor Responders to Follitropin Delta: A Retrospective Within-Patient Paired Study

**DOI:** 10.7759/cureus.108675

**Published:** 2026-05-11

**Authors:** Hiromasa Kuroda, Rina Nishida, Chisato Kuchihara, Kana Inukai, Koichiro Aoi, Masayo Yamada, Mariko Shindo, Naoko Murakami, Mika Koyama, Atsushi Haruki

**Affiliations:** 1 Obstetrics and Gynecology, Haruki Ladies Clinic, Osaka, JPN; 2 Embryology, Haruki Ladies Clinic, Osaka, JPN

**Keywords:** assisted reproductive technology (art), fertility, follitropin beta, follitropin delta, infertility, oocyte retrieval, ovarian stimulation, poor ovarian reserve, poor response, sterility

## Abstract

Background and objective

Follitropin delta (Rekovelle®) is administered using an individualized dosing algorithm based on anti-Müllerian hormone (AMH) and body weight, with a maximum dose of 12 μg/day in the first cycle. In patients who respond poorly to follitropin delta, alternative gonadotropins permitting higher dose administration, such as follitropin beta (Follistim®), may be considered. However, no within-patient study has examined whether switching to follitropin beta can rescue poor responders to follitropin delta. This study aims to evaluate whether switching to follitropin beta as a rescue protocol improves the number of oocytes retrieved and the quality of embryos in patients who responded poorly to follitropin delta, using a within-patient paired design.

Methods

This retrospective, single-center, within-patient paired study included patients who underwent follitropin delta antagonist-protocol cycles with letrozole co-administration and were subsequently switched to follitropin beta with letrozole co-administration due to poor ovarian response between January 2024 and April 2026. The last follitropin delta cycle, representing the poor-response cycle that triggered the treatment switch, and the first follitropin beta cycle per patient were analyzed as sequential matched pairs (n = 35). The primary outcome was the number of retrieved oocytes. Secondary outcomes included the number of metaphase II (MII) oocytes, transferable blastocysts, and high-quality blastocysts. The Wilcoxon signed-rank test was used. All follitropin delta cycles were administered at the maximum approved dose of 12 μg/day (approximately 180 IU), whereas all follitropin beta cycles were initiated at 300 IU/day.

Results

Median AMH was 0.64 ng/mL (interquartile range, 0.26-1.29), and median age was 40 years. In the subsequent follitropin beta cycles, the number of retrieved oocytes showed a trend toward an increase compared with the preceding follitropin delta cycles (median 6.0 (IQR 3.0-9.0) vs. 5.0 (IQR 3.0-7.0); p = 0.057; n = 35), with improvement in 19 of 35 patients. The number of MII oocytes was also numerically higher in follitropin beta cycles but did not reach statistical significance (median 5.0 (IQR 2.0-7.0) vs. 4.0 (IQR 2.0-5.0); p = 0.195). No significant differences were observed in transferable blastocysts (median 2.0 (IQR 0.5-4.0) vs. 2.0 (IQR 1.0-2.0); p = 0.123) or high-quality blastocysts (median 0.0 (IQR 0.0-1.0) vs. 0.0 (IQR 0.0-1.0); p = 0.060) between the two sequential cycles.

Conclusion

In patients who responded poorly to follitropin delta, switching to follitropin beta as a rescue protocol was associated with a trend toward increased oocyte retrieval, and no differences were observed in embryologic outcomes. These exploratory findings do not constitute evidence of a treatment benefit but may serve as a basis for hypothesis generation. Larger prospective studies are warranted.

## Introduction

Ovarian stimulation is a cornerstone of in vitro fertilization (IVF), with gonadotropin selection directly influencing treatment outcomes [1]. Follitropin delta (Rekovelle®, Ferring Pharmaceuticals), a recombinant follicle-stimulating hormone (FSH) produced in a human cell line (PER.C6®), employs an individualized dosing algorithm based on serum anti-Müllerian hormone (AMH) concentration and body weight. Phase III randomized controlled trials (RCTs) in Japanese and pan-Asian populations have demonstrated its non-inferiority to conventional gonadotropins for oocyte yield, alongside a significant reduction in ovarian hyperstimulation syndrome (OHSS) incidence [2,3]. Accordingly, follitropin delta has been adopted as the first-line gonadotropin at our center in Japan. However, unexpected poor ovarian responses to follitropin delta have been observed in a subset of patients at our institution (Haruki Ladies Clinic, Osaka, JPN), even among those expected to respond well based on AMH levels [4].

However, the maximum approved daily dose of follitropin delta is capped at 12 μg/day for the first treatment cycle in Japan, equivalent to approximately 180 IU of follicle-stimulating hormone (FSH) activity [5]. In patients with low AMH, this ceiling may result in insufficient follicular stimulation. By contrast, follitropin beta (Follistim®, Organon) permits administration at 300 IU/day or higher without a dose ceiling in poor responders, potentially delivering substantially greater exogenous FSH exposure. Furthermore, follitropin beta has a longer plasma half-life compared with follitropin alfa [6,7], which may sustain more consistent FSH receptor stimulation throughout the follicular phase.

Despite these pharmacological considerations, no head-to-head within-patient comparison of follitropin delta and follitropin beta has been reported in patients with a poor response to follitropin delta. Given the unmet clinical need to optimize gonadotropin protocols in poor responders [8], we conducted a retrospective within-patient paired study to evaluate whether a protocol change from follitropin delta to follitropin beta improves outcomes of ovarian stimulation in subsequent cycles.

## Materials and methods

Study design and participants

**Figure 1:**
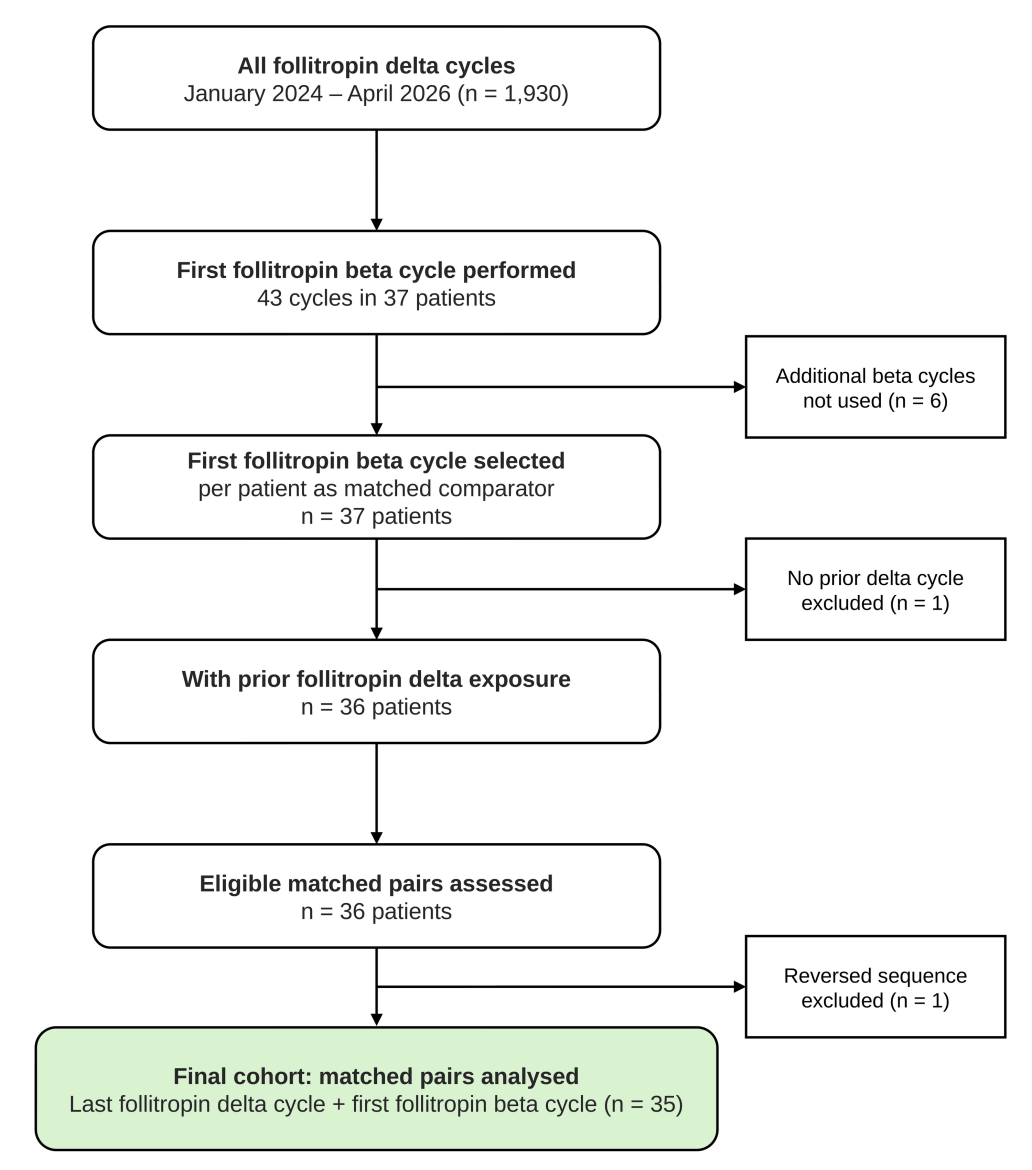
Flow diagram of the follitropin beta and delta (BELTA) study The diagram shows patient selection from 1,930 follitropin delta cycles to the final cohort of 35 matched pairs. AMH: Anti-Müllerian hormone; OPU: Oocyte pick-up

This is a single-center, retrospective, within-patient paired study conducted between January 2024 and April 2026. A total of 1,930 antagonist-protocol cycles using follitropin delta with letrozole co-administration and 43 antagonist-protocol cycles using follitropin beta with letrozole co-administration were performed at Haruki Ladies Clinic (Osaka, JPN). Of the 37 patients who underwent follitropin beta cycles, a total of 43 follitropin beta cycles were performed; six patients underwent more than one follitropin beta cycle, and only the first follitropin beta cycle per patient was used as the matched comparator. Of the 37 patients, 36 had also previously undergone at least one follitropin delta cycle at our clinic. One patient was excluded because the follitropin beta cycle preceded the follitropin delta cycle (reversed sequence), yielding a final cohort of 35 patients for analysis. The patient selection process is illustrated in Figure 1. The inclusion and exclusion criteria are summarized in Table 1. Poor ovarian response to follitropin delta was defined clinically as a retrieved oocyte count either below four or lower than anticipated based on the patient’s ovarian reserve markers (AMH and antral follicle count), as judged by the treating physician. This definition encompasses both the 'Patient-Oriented Strategies Encompassing Individualized Oocyte Number' (POSEIDON) criterion of fewer than four oocytes [9] and cases of suboptimal response in which the yield was disproportionately low relative to expected follicular output.

**Table 1:** Inclusion and exclusion criteria IVF: In vitro fertilization; ICSI: Intracytoplasmic sperm injection; OHSS: Ovarian hyperstimulation syndrome

Category	Criterion
Inclusion 1	Female patients undergoing IVF or intracytoplasmic sperm injection (ICSI)
Inclusion 2	At least one follitropin delta antagonist-protocol cycle with letrozole co-administration
Inclusion 3	At least one subsequent follitropin beta antagonist-protocol cycle following poor response to follitropin delta
Inclusion 4	Age 20 to 45 years
Exclusion 1	Cycles in which additional gonadotropin preparations were added to follitropin delta
Exclusion 2	Non-antagonist stimulation protocols (e.g., progestin-primed ovarian stimulation)
Exclusion 3	Prior ovarian surgery with potential impact on ovarian reserve
Exclusion 4	Cycle cancellation or protocol modification for OHSS prevention
Exclusion 5	Uterine fibroids or ovarian cysts precluding adequate transvaginal oocyte retrieval
Exclusion 6	Incomplete data precluding analysis of the primary outcome
Exclusion 7	Follitropin beta cycle not yet performed at the time of data extraction

This study was approved by the Institutional Review Board of Haruki Ladies Clinic (approval no. 2026-02) and conducted in accordance with the Declaration of Helsinki. Blanket informed consent covering non-study-specific research was obtained from all patients. Additionally, an opt-out opportunity was provided via notice posted on our clinic's website.

Stimulation protocols

All cycles used a flexible multiple-dose gonadotropin-releasing hormone (GnRH) antagonist protocol with letrozole co-administration, an approach reported to reduce gonadotropin requirements in poor responders [10]. For follitropin delta cycles, dosing was determined by the manufacturer's AMH- and weight-based algorithm (maximum 12 μg/day for the first cycle). For follitropin beta cycles, all patients were initiated at 300 IU/day, with subsequent dose adjustments made according to follicular response at the attending physician's discretion. A GnRH antagonist (ganirelix (Ganirelix®) or cetrorelix (Cetrotide®)) was introduced when the leading follicle reached ≥14 mm. Based on the number of follicles and/or serum estradiol levels, oocyte maturation was triggered with human chorionic gonadotropin (hCG) administration (choriogonadotropin alfa 250 μg (Ovidrel®) or urinary hCG 10,000 IU) and/or a GnRH agonist when the leading follicle reached ≥17 mm. Oocyte retrieval was performed 38 to 40 hours after trigger.

Embryo culture and cryopreservation

Retrieved oocytes were subjected to conventional IVF or intracytoplasmic sperm injection (ICSI). The ICSI was performed for all oocytes in cases of severe male factor infertility, positive sperm immobilization antibody, or a history of fertilization failure in a prior cycle. In cases without these indications, a split insemination approach was used, in which half of the mature oocytes underwent conventional IVF, and the remainder underwent ICSI; if conventional IVF demonstrated superior fertilization in a prior cycle, all oocytes were inseminated by conventional IVF. All fertilized oocytes were cultured to the blastocyst stage on day five or day six; no cleavage-stage embryos were cryopreserved. Blastocysts meeting the transfer eligibility criteria were cryopreserved using the vitrification method for subsequent frozen-thawed embryo transfer.

Outcome measures

The primary outcome was the number of retrieved oocytes per cycle. Secondary outcomes included the number of metaphase II (MII) oocytes, total transferable blastocysts, and high-quality blastocysts. Blastocyst quality was assessed using the Gardner grading system [11]; blastocysts eligible for transfer were defined as those with a blastocoel expansion grade of ≥3 on day five or day six and an inner cell mass (ICM) grade other than C, and blastocysts graded ≥3BB were classified as high-quality. Cycles yielding zero blastocysts were recorded as zero.

Statistical analysis

For each patient, the last follitropin delta cycle and the first subsequent follitropin beta cycle were selected as the sequential matched pair. Continuous variables are reported as median (interquartile range (IQR)). The Wilcoxon signed-rank test was used to compare paired outcomes between the two sequential cycles [12]. A two-sided p-value < 0.05 was considered statistically significant. All analyses were performed using R version 4.5.3 (R Foundation for Statistical Computing, Vienna, AUT).

## Results

Patient characteristics

A total of 35 sequential matched pairs were included in the final analysis. Patient baseline characteristics are summarized in Table 2. Median age was 40 years (IQR 36-41), and median AMH was 0.64 ng/mL (IQR 0.26-1.29), confirming that the cohort predominantly comprised patients with diminished ovarian reserve. All patients were initiated at 300 IU/day follitropin beta in their subsequent cycles, compared with the algorithm-determined maximum dose of 12 μg/day follitropin delta in all preceding cycles.

**Table 2:** Patient baseline characteristics (total n = 35) IQR: Interquartile range; AMH: Anti-Müllerian hormone; OPU: Oocyte pick-up

Characteristic	Value
Age at OPU (years), median (IQR)	40.0 (36.0-41.0)
AMH (ng/mL), median (IQR)	0.64 (0.260-1.29)
BMI (kg/m²), median (IQR)	22.6 (19.6-24.6)
Prior OPU cycles (n), median (IQR)	3.0 (2.0-4.0)
Infertility duration (months), median (IQR), n = 34	14.0 (7.0-23.2)
Follitropin beta total dose (IU), median (IQR)	3,600 (2,775-4,350)
Follitropin beta stimulation days, median (IQR)	11 (10-12)
Follitropin delta starting dose (μg/day), median (IQR)	12 (12-12)
Follitropin delta total dose (μg), median (IQR)	108 (84-138)
Follitropin delta stimulation days, median (IQR)	10 (8-11)

Primary outcome (retrieved oocytes)

**Figure 2:**
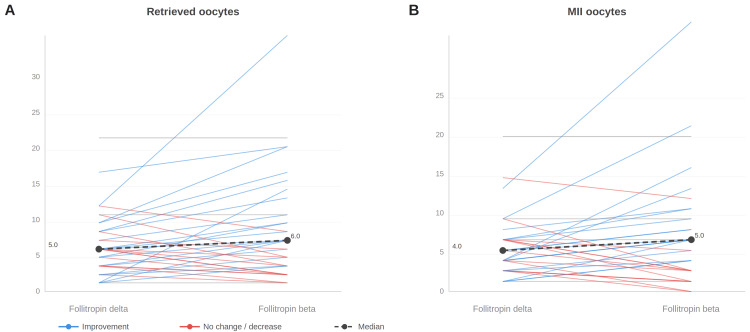
Paired comparison of oocyte outcomes Panel A shows a patient-level paired plot for retrieved oocytes, while panel B depicts a patient-level paired plot for MII oocytes, comparing follitropin delta and follitropin beta cycles in 35 patients. Blue lines indicate an increase in the follitropin beta cycle compared with the preceding follitropin delta cycle, and red lines indicate no change or a decrease. Dashed lines represent medians. MII: Metaphase II

In the subsequent follitropin beta cycles, the number of retrieved oocytes showed a trend toward an increase compared with the preceding follitropin delta cycles (median 6.0 (IQR 3.0-9.0) vs. 5.0 (IQR 3.0-7.0); Δ median +1.0; p = 0.057; n = 35). Of the 35 patients, 19 (54.3%) showed an increase in oocyte yield in their follitropin beta cycles, 13 (37.1%) showed a decrease, and three (8.6%) showed no change (Table 3). Patient-level paired data for retrieved oocytes and MII oocytes are shown in Figure 2.

**Table 3:** Within-patient comparison of ovarian stimulation outcomes between sequential cycles (total n = 35) IQR: Interquartile range

Outcome	Preceding follitropin delta cycles median (IQR)	Subsequent follitropin beta cycles median (IQR)	Δ median	p-value	Improved/decreased/unchanged
Retrieved oocytes	5.0 (3.0-7.0)	6.0 (3.0-9.0)	1.0	0.057	19/13/3
MII oocytes	4.0 (2.0-5.0)	5.0 (2.0-7.0)	1.0	0.195	18/13/4
Transferable blastocysts	2.0 (1.0-2.0)	2.0 (0.5-4.0)	0.0	0.123	17/11/7
High-quality blastocysts	0.0 (0.0-1.0)	0.0 (0.0-1.0)	0.0	0.060	10/5/20

Secondary outcomes

The number of MII oocytes was numerically higher in the follitropin beta cycles but did not reach statistical significance (median 5.0 (IQR 2.0-7.0) vs. 4.0 (IQR 2.0-5.0); p = 0.195; n = 35). The number of transferable blastocysts was comparable between the two sequential cycles (median 2.0 (IQR 0.5-4.0) vs. 2.0 (IQR 1.0-2.0); p = 0.123; n = 35). The number of high-quality blastocysts was similarly comparable between the two cycles (median 0.0 (IQR 0.0-1.0) vs. 0.0 (IQR 0.0-1.0); p = 0.060; Hodges-Lehmann estimate: 0.0, 95% confidence interval: 0.0 to 0.5).

## Discussion

To our knowledge, this is the first within-patient comparison of follitropin delta and follitropin beta in patients who responded poorly to the former during the antagonist protocol for IVF/ICSI. Our findings demonstrate a trend toward improved oocyte retrieval in subsequent follitropin beta cycles (p = 0.057), with more than half of patients showing increased yield in their follitropin beta cycle than in their preceding follitropin delta cycle. However, this trend did not reach statistical significance, and no significant improvement was observed in transferable blastocyst number or quality.

The biological rationale for expecting benefit from follitropin beta in this setting rests on two pharmacological considerations. First, the maximum approved dose of follitropin delta in the first cycle is capped at 12 μg/day (approximately 180 IU) [5], whereas follitropin beta can be initiated at 300 IU/day, providing approximately 1.7-fold greater FSH exposure. This dose advantage may be sufficient to recruit additional follicles in patients at the threshold of gonadotropin responsiveness. Subsequently, follitropin beta has a longer plasma half-life compared with follitropin alpha [6,7], potentially sustaining more consistent follicular stimulation. Whether these advantages translate clinically with follitropin delta, which itself has a distinct glycosylation profile and pharmacokinetic properties derived from its human cell line origin [13], warrants further investigation. It should be noted that this study compares a dose-capped follitropin delta regimen with a higher-dose follitropin beta regimen; therefore, the observed differences reflect the combined effect of gonadotropin type and dosage, and the contribution of each cannot be separated in this design.

The absence of improvement in blastocyst development despite the trend in oocyte retrieval is clinically important. Higher exogenous FSH doses may affect granulosa cell function and oocyte developmental competence. The low AMH of this cohort (median 0.64 ng/mL) also indicates an intrinsically compromised oocyte pool. Additionally, the marked heterogeneity of individual responses, ranging from +20 to −5 retrieved oocytes, highlights the existence of two distinct patient subgroups: those who respond favorably to the protocol change and those who do not. Although no clinical or laboratory parameters currently allow prospective identification of these subgroups, this observation underscores the importance of identifying predictive biomarkers, such as FSH receptor (FSHR) polymorphisms or antral follicle count (AFC), to guide individualized gonadotropin selection in poor responders [14]. These findings are consistent with data showing that higher follitropin delta doses in subsequent cycles improve oocyte yield without necessarily improving blastocyst numbers [15]. These findings also raise important questions regarding the relationship between FSH dose and oocyte quality. Several lines of evidence suggest that higher FSH doses, while potentially increasing oocyte numbers, may paradoxically compromise oocyte developmental competence. A large-scale analysis of over 650,000 assisted reproductive technology (ART) cycles demonstrated a negative correlation between total FSH dose and retrieved oocyte number above an optimal threshold, challenging the assumption that greater FSH exposure invariably yields more oocytes [16]. Prospective randomized studies have shown that higher FSH doses increase oocyte yield but reduce the proportion of high-quality and euploid blastocysts, such that absolute blastocyst numbers remain comparable to lower-dose protocols [17,18]. Notably, within the follitropin delta dose-response data, fertilization rate and blastocyst-to-oocyte ratio decreased significantly with increasing doses in both low- and normal-AMH strata, despite more oocytes being retrieved at higher doses [15]. Furthermore, follitropin delta, despite delivering a lower FSH exposure than the 300 IU/day follitropin beta used in the subsequent cycles of the present study, achieved comparable oocyte yields and clinical pregnancy rates to conventional follitropin alfa or beta in large RCTs [2,3,19]. Taken together, these data suggest that in poor responders, the additional FSH exposure provided by follitropin beta may increase oocyte retrieval in some patients but may not translate into proportional improvements in oocyte quality or blastocyst yield, a pattern consistent with the present observations.

The within-patient paired study design substantially mitigates confounding inherent to conventional between-group comparisons; a similar self-paired approach has been used previously to evaluate letrozole co-administration in poor responders [20]. By using each patient as their own control across sequential cycles, differences in ovarian reserve, age, and uterine factors are inherently controlled. This design is particularly suited to the present setting, in which all follitropin beta cycles followed prior follitropin delta cycles in the same patients.

A further consideration is the possibility of regression to the mean. Because patients were switched to follitropin beta specifically after a poor response to follitropin delta, the reference cycle was, by definition, a relatively low-performing cycle for each patient. Some of the apparent improvement observed in the subsequent follitropin beta cycles may therefore reflect natural cycle-to-cycle variation in ovarian response rather than a true pharmacological advantage of follitropin beta. Individual oocyte yield is known to vary substantially between cycles, even with the same gonadotropin protocol, and a proportion of patients who performed poorly in one cycle would be expected to improve in the next, regardless of treatment change. While the within-patient paired design partially mitigates between-patient confounding, it cannot eliminate this source of bias. This limitation reinforces the need for a prospective randomized design in future studies, in which allocation to follitropin beta is not contingent on prior poor response. It is noteworthy, however, that the follitropin beta cycles were performed after the follitropin delta cycles in all patients, meaning that ovarian reserve had, if anything, further declined by the time follitropin beta was administered. The temporal disadvantage faced by follitropin beta in this study design suggests that the observed trend toward improved oocyte retrieval may, if real, actually underestimate the true pharmacological advantage of follitropin beta.

This study has several important limitations that should be considered when interpreting the findings. First, the retrospective single-center design introduces inherent selection bias and limits generalizability to other clinical settings. Second, the sample size of 35 pairs provides limited statistical power; a formal power calculation suggests that approximately 50 pairs would be required to achieve 80% power to detect a median difference of two oocytes, and the present cohort is likely underpowered to draw definitive conclusions. The borderline p-values observed (p = 0.057 for retrieved oocytes, p = 0.060 for high-quality blastocysts) should be interpreted with caution, as they do not constitute statistically significant evidence, and the true treatment effect, if any, remains uncertain. Third, the interval between the preceding follitropin delta cycle and the subsequent follitropin beta cycle varied considerably across patients. Any interval-related decline in ovarian reserve, which is particularly pronounced in poor responders with low AMH, could confound the within-patient comparison and artificially reduce the apparent benefit of follitropin beta. The possibility of regression to the mean and natural cycle-to-cycle variation as alternative explanations for the observed improvement is discussed above. Fourth, cycle-level AMH values were not systematically available, precluding adjustment for interval changes in ovarian reserve. Fifth, the contribution of sequential cycle learning, whereby stimulation protocols are refined based on prior cycle experience, cannot be excluded as a confounding factor. The improvement observed in follitropin beta cycles may partly reflect protocol optimization rather than a true pharmacological advantage of follitropin beta per se. Sixth, the dose of follitropin beta was fixed at 300 IU/day as the starting dose for all patients; whether higher starting doses would yield further benefit remains unknown. Seventh, blastocyst grading was performed at a single center by a limited number of embryologists, and interobserver variability in quality assessment may have affected the secondary endpoints. Eighth, this study did not assess cumulative live birth rates, which represent the most clinically meaningful outcome in ART.

The absence of significant improvement in blastocyst endpoints in this pilot study does not exclude a clinically meaningful benefit in ongoing or cumulative pregnancy rates. In summary, this study should be regarded as a pilot study. The findings are insufficient to support definitive clinical recommendations [21], and a prospective randomized or well-matched cohort study with prespecified stratification by AMH, AFC, and FSHR genotype is warranted to confirm or refute these observations [8].

## Conclusions

In patients who demonstrated poor ovarian response to follitropin delta, switching to follitropin beta as a rescue protocol was associated with a trend toward increased oocyte retrieval, while embryologic outcomes did not improve. These findings suggest that a gonadotropin permitting flexible high-dose administration may have potential as a rescue option in this difficult-to-treat population. As these findings are exploratory, larger prospective studies are warranted to confirm them and identify patients most likely to benefit.
